# Multiple positron emission tomography tracers for use in the classification of gliomas according to the 2016 World Health Organization criteria

**DOI:** 10.1093/noajnl/vdaa172

**Published:** 2020-12-07

**Authors:** Keisuke Miyake, Kenta Suzuki, Tomoya Ogawa, Daisuke Ogawa, Tetsuhiro Hatakeyama, Aya Shinomiya, Nobuyuki Kudomi, Yuka Yamamoto, Yoshihiro Nishiyama, Takashi Tamiya

**Affiliations:** 1 Department of Neurological Surgery, Kagawa University, Faculty of Medicine, Ikenobe, Miki-Cho, Kita-gun, Kagawa, Japan; 2 Department of Medical Physics, Kagawa University, Faculty of Medicine, Ikenobe, Miki-Cho, Kita-gun, Kagawa, Japan; 3 Department of Radiology, Kagawa University, Faculty of Medicine, Ikenobe, Miki-Cho, Kita-gun, Kagawa, Japan

**Keywords:** 2016 World Health Organization classification, glioma, positron emission tomography

## Abstract

**Background:**

The molecular diagnosis of gliomas such as isocitrate dehydrogenase (IDH) status (wild-type [wt] or mutation [mut]) is especially important in the 2016 World Health Organization (WHO) classification. Positron emission tomography (PET) has afforded molecular and metabolic diagnostic imaging. The present study aimed to define the interrelationship between the 2016 WHO classification of gliomas and the integrated data from PET images using multiple tracers, including ^18^F-fluorodeoxyglucose (^18^F-FDG), ^11^C-methionine (^11^C-MET), ^18^F-fluorothymidine (^18^F-FLT), and ^18^F-fluoromisonidazole (^18^F-FMISO).

**Methods:**

This retrospective, single-center study comprised 113 patients with newly diagnosed glioma based on the 2016 WHO criteria. Patients were divided into 4 glioma subtypes (Mut, Codel, Wt, and glioblastoma multiforme [GBM]). Tumor standardized uptake value (SUV) divided by mean normal cortical SUV (tumor–normal tissue ratio [TNR]) was calculated for ^18^F-FDG, ^11^C-MET, and ^18^F-FLT. Tumor–blood SUV ratio (TBR) was calculated for ^18^F-FMISO. To assess the diagnostic accuracy of PET tracers in distinguishing glioma subtypes, a comparative analysis of TNRs and TBR as well as the metabolic tumor volume (MTV) were calculated by Scheffe's multiple comparison procedure for each PET tracer following the Kruskal–Wallis test.

**Results:**

The differences in mean ^18^F-FLT TNR and ^18^F-FMISO TBR were significant between GBM and other glioma subtypes (*P* < .001). Regarding the comparison between Gd-T1WI volumes and ^18^F-FLT MTVs or ^18^F-FMISO MTVs, we identified significant differences between Wt and Mut or Codel (*P* < .01).

**Conclusion:**

Combined administration of 4 PET tracers might aid in the preoperative differential diagnosis of gliomas according to the 2016 WHO criteria.

Key Points

- Usefulness of 4 PET tracers for glioma classification based on 2016 WHO criteria.
- Comparison between Gd-T1WI volume and MTV of ^18^F-FLT or ^18^F-FMISO was effective to classify between Wt and Mut or Codel.

**Importance of the Study**

This is the first study examining the relationship between the 2016 WHO glioma classification and glioma classification based on multiple PET tracers to evaluate different metabolic pathways, including glucose, amino acid, and nucleic acid metabolism, and the presence of hypoxic regions. The differences in mean ^18^F-FLT TNR and ^18^F-FMISO TBR were significant between GBM and other glioma subtypes. The differences in mean ^11^C-MET TNR were significant between GBM and Mut or Wt. There were significant differences in the MTV of ^18^F-FLT between GBM and Mut or Codel. A comparison between Gd-T1WI volume and the MTV of ^11^C-MET was significant between GBM and Codel or Wt. A comparison between Gd-T1WI volume and the MTV of ^18^F-FLT or ^18^F-FMISO revealed significant differences between Wt and Mut or Codel. We suggest that multiple PET tracers using ^18^F-FDG, ^11^C-MET, ^18^F-FLT, and ^18^F-FMISO are useful for preoperative differential diagnosis of gliomas.

According to the 2007 World Health Organization (WHO) grading criteria, gliomas, the most common primary brain tumors, comprise a heterogeneous group of histological subtypes based on cellular alterations related to tumor aggressiveness.^1^ Additionally, the 2016 WHO classification of Central Nervous System (CNS) tumors includes molecular genetic profiles for the subclassification of gliomas.^2^ Mutations in coding sequences of isocitrate dehydrogenase (*IDH*) *1* and *IDH2* and chromosome 1p and 19q (1p19q) codeletion are essential for the diagnosis of gliomas reclassified as astrocytic and oligodendroglial tumors.^3,4^ Surgical specimens are indispensable for the definitive molecular pathological diagnosis according to the 2016 WHO criteria. However, in some patients, glioma localization hinders sample collection for pathological assessment and preoperative methods that can predict the glioma genotype are necessary for determining treatment strategies.

Diagnostic imaging of gliomas achieved through various methods has significantly advanced over recent years. Magnetic resonance imaging (MRI), the most commonly used method to collect information on tumor morphology, cannot by itself determine the definitive diagnosis. Conversely, positron emission tomography (PET) has facilitated the establishment of noninvasive metabolic and molecular imaging methods for CNS tumors. Importantly, various molecular processes can be visualized using specific PET tracers. Among these, ^18^F-fluorodeoxyglucose (^18^F-FDG), the most frequently used radiotracer, is a glucose analog whose metabolism involves glucose transporter and hexokinase activity. Additionally, several other PET tracers for CNS tumors have been developed based on the key roles of certain amino acids such as ^11^C-methionine (^11^C-MET),^5^  ^18^F-fluoroethyltyrosine (^18^F-FET),^6^ and ^18^F-fluorodopa (^18^F-FDOPA).^7^  ^11^C-MET is used to evaluate protein synthesis and cell proliferation in gliomas and to detect malignant transformation.^8^  ^18^F-fluorothymidine (^18^F-FLT) is a radiolabeled thymidine analog used to predict tumor progression,^9^ and provides a low background, facilitating tumor detection.^10^ Malignant tumors are characterized by a hypoxic tissue environment which may drive peripheral tumor growth and is associated with tumor progression. One of the most widely used PET tracers for molecular imaging of hypoxia is ^18^F-fluoromisonidazole (^18^F-FMISO).^11^

We previously reported the characteristics of gliomas based on the 2007 WHO criteria using ^18^F-FDG, ^11^C-MET, ^18^F-FLT, and ^18^F-FMISO PET.^8^ However, these results might be impacted with the addition of genetic information such as *IDH* status (wild-type [wt] or mutation [mut]) in the 2016 WHO glioma classification. The present study aimed to define the interrelationship between gliomas classified according to the 2016 WHO criteria and the integrated data from multiple PET studies evaluating the metabolism of ^18^F-FDG, ^11^C-MET, ^18^F-FLT, and ^18^F-FMISO.

## Materials and Methods

### Patients

This retrospective, single-center study complied with the precepts established by the Declaration of Helsinki and was approved by the Kagawa University Faculty of Medicine Human Subjects Ethics Committee (no. 2019–027). ^18^F-FDG, ^11^C-MET, ^18^F-FLT, and ^18^F-FMISO were approved for use as PET tracers by the Kagawa University Faculty of Medicine Human Subjects Ethics Committee, and an informed written consent was obtained from all participants.

From April 2009 to March 2019, 130 patients underwent ^18^F-FDG, ^11^C-MET, ^18^F-FLT, and ^18^F-FMISO PET evaluation at Kagawa University Faculty of Medicine in Japan. We included 113 patients in the final diagnosis after excluding those who were not assessed by all 4 PET tracers, did not undergo histopathological and molecular analyses, and were diagnosed with not-otherwise-specified lesions (Table 1).^2^

**Table 1.** Patient Characteristics in 4 Glioma Subtypes

Subtype	No. of Patients	F/M	Mean age ± SD (min–max) (years)
**Mut**	**22**	**14/8**	**37.7 ± 9.9 (21–55)**
DA *IDH-*mut	8	8/0	37.6 ± 9.5 (27–50)
AA *IDH*-mut	14	6/8	37.7 ± 10.5 (21–55)
**Codel**	**14**	**9/5**	**48.3 ± 15.0 (28–71)**
OD	6	5/1	42.2 ± 10.1 (31–60)
AO	8	4/4	52.9 ± 17.0 (28–71)
**Wt**	**14**	**6/8**	**63.1 ± 13.3 (35–79)**
DA *IDH-*wt	5	1/4	58.6 ± 13.6 (35–69)
AA *IDH*-wt	9	5/4	65.7 ± 13.3 (35–79)
**GBM**	**63**	**31/32**	**63.7 ± 14.8 (26–86)**
GBM *IDH*-mut	5	2/3	37.4 ± 6.7 (27–45)
GBM *IDH*-wt	58	29/29	66.0 ± 13.0 (26–86)

Following 4 glioma subtypes; Mut, Codel, Wt, and GBM.

Mut; DA IDH-mut and AA IDH-mut, Codel; OD and AO, Wt; DA IDH-wt and AA IDH-wt, GBM; GBM IDH-mut and GBM IDH-wt.

AA, anaplastic astrocytoma; AO, anaplastic oligodendroglioma; DA, diffuse astrocytoma; GBM, glioblastoma multiforme; IDH, isocitrate dehydrogenase; mut, mutation; OD, oligodendroglioma; PET, positron emission tomography; wt, wild type.

According to the 2016 WHO criteria,^2^ tumors were classified as diffuse astrocytoma (DA) with isocitrate dehydrogenase *(IDH)1/2* mutation (mut) without 1p19q codeletion (DA *IDH-*mut), anaplastic astrocytoma (AA) with *IDH1/2*-mut without 1p19q codeletion (AA *IDH*-mut), oligodendroglioma (OD) with *IDH1/2*-mut and 1p19q codeletion, anaplastic oligodendroglioma (AO) with *IDH1/2*-mut and 1p19q codeletion, DA with *IDH1/2* wild type (wt) (DA *IDH-*wt), AA with *IDH1/2* wt (AA *IDH*-wt), glioblastoma multiforme (GBM) with *IDH1/2*-mut (GBM *IDH*-mut), and GBM with *IDH1/2* wt (GBM *IDH*-wt). In this study, tumors were divided into Mut, Codel, Wt, and GBM glioma subtypes and were evaluated as follows: Mut, DA *IDH*-mut and AA *IDH*-mut; Codel, OD and AO; Wt, DA *IDH*-wt and AA *IDH*-wt; and GBM, GBM *IDH*-mut and GBM *IDH*-wt. All included patients were orally informed with the details regarding the study and provided their informed consent.

### Histopathological and Molecular Analyses

To reclassify the study cohort according to the 2016 WHO classification, the study patients were evaluated for *IDH*-mut and 1p19q codeletion. For *IDH*-mut status, *IDH*1^R132H^ protein expression was determined by immunohistochemistry using a monoclonal antibody (clone H09, 1:50; Dianova, Germany). In cases where immunostaining was not possible, *IDH1* (R132) and *IDH2* (R172) were directly sequenced using the Sanger method. The 1p19q codeletion status was analyzed by fluorescence *in situ* hybridization with locus-specific probes for 1p36 and 19q13.

### MRI and PET

MRI was performed on a 3-T MAGNETOM Skyra (Siemens Healthcare, Erlangen, Germany) scanner. T2-weighted axial fluid-attenuated inversion recovery images (FLAIR) (repetition time/echo time, 10 000/93 ms; slice, 5 mm; matrix, 224 × 320), gadolinium-contrast axial T1-weighted images (Gd-T1WI) (repetition time/echo time, 400/11 ms; slice, 5 mm; matrix, 230 × 384) and diffuse weighted imaging (DWI) (repetition time/echo time, 5300/69 ms; slice, 5 mm; matrix, 160 × 160; 1000 s/mm^2^ B-value) were acquired.

PET studies were performed using a Biograph mCT PET/CT scanner (Siemens Medical Solutions Knoxville, TN, USA). PET scans were acquired in the three-dimensional model, and PET images were reconstructed as described in our previous study (the simultaneous acquisition of 51 transverse images per field of view [FOV], with an intersection spacing of 3 mm, for a total axial FOV of 15 cm).^10^ PET radiotracers were produced using an HM-18 cyclotron (Sumitomo Heavy Industries, Tokyo, Japan). The radiochemical purity of ^11^C-MET,^12^  ^18^F-FLT,^13^ and ^18^F-FMISO^14^ were >95%. Transmission and regional emission images of the brain were obtained as described in our previous study.^10^ Fasting was initiated 6 h before all PET studies, and the examination schedule was as follows: MRI, including contrast examination, was performed on day 1, ^18^F-FMISO was performed on day 2, ^18^F-FLT was performed on day 3, and ^11^C-MET was performed on the morning of day 4, followed by ^18^F-FDG during the afternoon of day 4.

### Image Analyses

The uptake of ^18^F-FDG, ^11^C-MET, and ^18^F-FLT in brain tumors were semiquantitatively assessed by obtaining the standardized uptake values (SUVs). A region of interest around the hottest portion of each lesion was manually set by an observer. The maximum SUV (SUV_max_) was considered as the representative value for each tumor. The maximum tumor-to-normal ratio (TNR) was determined by dividing the tumor SUV_max_ by the mean SUV of the normal brain parenchyma (usually contralateral normal cerebral tissue excluding the ventricles). The uptake of ^18^F-FMISO in the brain tumor was semiquantitatively assessed by evaluating the SUV_max_. The ^18^F-FMISO PET images were converted into average venous blood concentration of ^18^F-FMISO to obtain the tumor-to-blood ratios (TBRs), allowing for a three-dimensional pixel-by-pixel calculation of the maximum TBR for SUV_max_. The tumor volumes were measured by performing a three-dimensional, threshold-based, volume-of-interest analysis of the hyperintensity on fluid-attenuated inversion recovery (FLAIR) images, hyperintensity on diffusion-weighted images (DWI), and contrast-enhanced lesions on gadolinium-enhanced T1-weighted images (Gd-T1WI). For PET studies, the cutoff values of 1.1 on the ^18^F-FDG TNR, 1.3 on the ^11^C-MET TNR, 1.3 on the ^18^F-FLT TNR, and 1.2 on the ^18^F-FMISO TBR were used to determine the metabolic tumor volume (MTV).^8,15^ The PET and MRI datasets were transferred to a Linux workstation, and coregistration of ^18^F-FDG/^11^C-MET/^18^F-FLT/^18^F-FMISO/MRI was performed using Dr. View/Linux, version R2.5 (AJS, Tokyo, Japan). Before the histopathological and molecular diagnoses, 2 radiologists (Y. Y. and Y. N.) analyzed the data to lower the risk of observer bias to the maximum extent possible.

### Statistical Analysis

The relationship of glioma subtypes with the volume on FLAIR, Gd-T1WI, and DWI, mean TNRs on ^18^F-FDG, ^11^C-MET, and ^18^F-FLT, mean TBR on ^18^F-FMISO, MTV on 4 PET studies were examined. To assess the diagnostic accuracy of PET tracers in distinguishing glioma subtypes, a comparative analysis of TNRs and TBR as well as the MTV were calculated by Scheffe's multiple comparison procedure of each PET tracer following the Kruskal–Wallis test. All parametric data were expressed as averages with standard deviation. Differences were considered statistically significant at a *P* value of <.05. The cutoff values for volume on FLAIR, Gd-T1WI, and DWI, mean TNRs on ^18^F-FDG, ^11^C-MET, and ^18^F-FLT, mean TBR on ^18^F-FMISO, MTV on 4 PET studies in receiver operating characteristic (ROC) curve, area under the curve (AUC), sensitivity, specificity, odds ratios (ORs), 95% Confidence interval (CI), and *P* value by the log-rank test at the cutoff value were compared and examined between glioma subtypes. The cutoff values with the highest sensitivity and specificity were used. Similarly, the MTV of each PET tracer and the volume of each MRI were compared (MTV of PET divided by volume of MRI) and examined. All statistical analyses were performed using the SPSS statistical software package (version 26; IBM).

## Results

### Patient Characteristics

Table 1 summarizes the characteristics of 113 patients (median age, 56.7 [21–86] years; 60 females and 53 males) classified into Mut (22 cases), Codel (14 cases), Wt (14 cases), and GBM (63 cases) for glioma subtypes.

### Correlation of Glioma Subtypes with TNR and TBR Values

**Figure 1.**
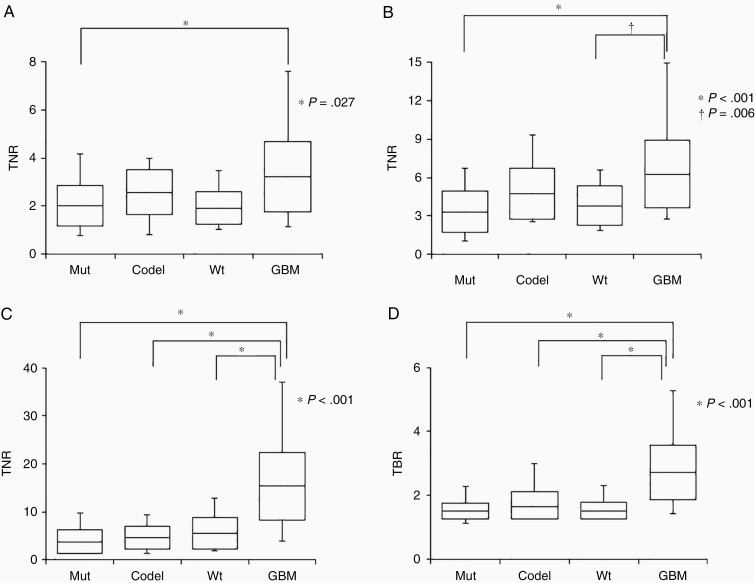
Box plots indicate the TNRs of ^18^F-FDG) (A), ^11^C-MET (B), and^18^F-FLT (C) and TBR of ^18^F-FMISO (D) for 4 glioma subtypes. Lines within the boxes indicate the average boxes represent standard deviation, and whiskers denote minimum–maximum.

Figure 1 shows the correlation of glioma subtypes with the ^18^F-FDG, ^11^C-MET, and ^18^F-FLT TNRs and ^18^F-FMISO TBR. The mean ^18^F-FDG TNRs were 2.02 ± 0.84, 2.58 ± 0.92, 1.92 ± 0.67, and 3.22 ± 1.47 for Mut, Codel, Wt, and GBM, respectively. The differences in ^18^F-FDG TNRs between GBM and Mut were statistically significant (*P* = .027) (Figure 1A). The mean ^11^C-MET TNRs for Mut, Codel, Wt, and GBM were 3.32 ± 1.64, 4.74 ± 1.98, 3.79 ± 1.54, and 6.27 ± 2.66, respectively. The differences in mean ^11^C-MET TNRs were significant between GBM and Mut (*P* < .001) and GBM and Wt (*P* = .006) (Figure 1B). The cutoff value of ^11^C-MET TNRs was 4.424 between GBM and Mut or 4.327 between GBM and Wt. The mean ^18^F-FLT TNRs for Mut, Codel, Wt, and GBM were 3.75 ± 2.47, 4.69 ± 2.39, 5.61 ± 3.31, and 15.41 ± 7.03, respectively. The differences in mean ^18^F-FLT TNRs between GBM and other glioma subtypes were significant (*P* < .001) (Figure 1C). The cutoff value of ^18^F-FLT TNRs was 6.455 between GBM and Mut, 6.389 between GBM and Codel, and 7.563 between GBM and Wt. The mean ^18^F-FMISO TBRs for Mut, Codel, Wt, and GBM were 1.51 ± 0.24, 1.66 ± 0.45, 1.52 ± 0.28, and 2.71 ± 0.85, respectively. The differences in ^18^F-FMISO TBRs were statistically significant between GBM and other glioma subtypes (*P* < .001; Figure 1D). The cutoff value of ^18^F-FLT TNRs was 1.760 between GBM and Mut, 1.875 between GBM and Codel, and 1.612 between GBM and Wt. (see Supplementary Table 2)

### Correlation of Glioma Subtypes with Volume of MRI and MTVs of 4 PET Tracers

**Figure 2.**
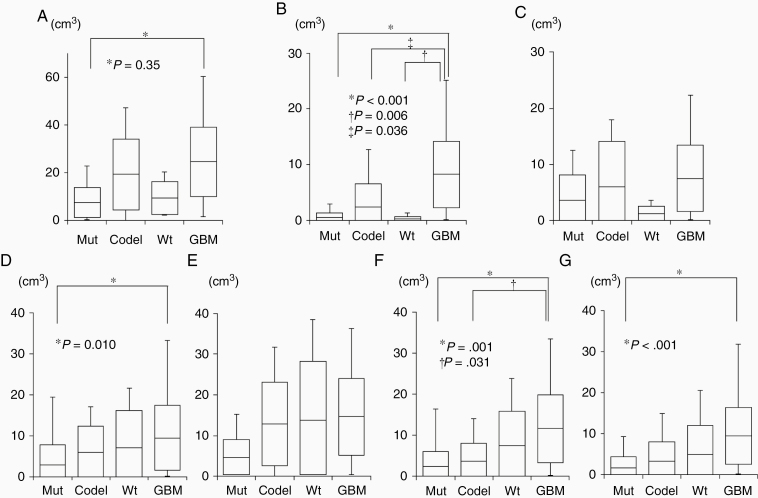
Box plots indicate the volumes of FLAIR (A), Gd-T1WI (B), DWI (C), and MTVs of ^18^F-FDG (D), ^11^C-MET (E), ^18^F-FLT (F), and ^18^F-FMISO (G) for 4 glioma subtypes. Lines within the boxes indicate the average, boxes represent standard deviation, and whiskers denote minimum–maximum.

Figure 2 shows the correlation of glioma subtypes with the volumes of FLAIR, Gd-T1WI, and DWI and MTVs of ^18^F-FDG, ^11^C-MET, and ^18^F-FLT, and ^18^F-FMISO.

***Correlations between glioma subtypes and MRI volumes***There was a significant difference in FLAIR volumes between Mut (7.42 ± 6.25 cm^3^) and GBM (24.55 ± 14.38 cm^3^, *P* = .035; Figure 2A). Gd-T1WI volumes were significantly different between GBM (8.28 ± 5.95 cm^3^) and Mut (0.51 ± 0.87 cm^3^, *P* < .001), Codel (0.82 ± 1.25 cm^3^, *P* =.036), and Wt (0.28 ± 0.38 cm^3^, *P* = .006; Figure 2B). DWI volumes were not significantly different among the glioma subtypes (Figure 2C;Supplementary Table 1)

***Correlations between glioma subtypes and MTVs of 4 PET tracers*** ^18^F-FDG MTVs were significantly different between Mut (2.55 ± 4.27 cm^3^) and GBM (9.47 ± 7.91 cm^3^) (*P* = .010) (Figure 2D). ^11^C-MET MTVs were not significantly different among the glioma subtypes (Figure 2E). ^18^F-FLT MTVs were significantly different between GBM (11.59 ± 8.35 cm^3^) and Mut (2.42 ± 3.78 cm^3^, *P* = .001) and between GBM and Codel (3.78 ± 4.75 cm^3^, *P* = .031; Figure 2F). ^18^F-FMISO MTVs were significantly different between Mut (1.54 ± 2.27 cm^3^) and GBM (9.58 ± 7.04 cm^3^, *P* < .001; Figure 2G;Supplementary Table 2).

#### Correlations among glioma subtypes with the comparison between MTVs of 4 PET tracers and volume of each MRI

##### Correlations Among Glioma Subtypes with the Comparison Between MTVs of 4 PET Tracers and FLAIR Volumes. 

**Figure 3.**
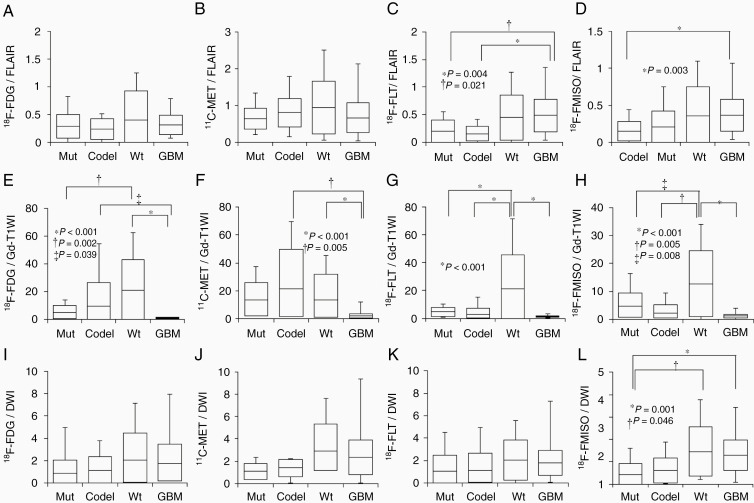
Box plots indicating the comparisons between the FLAIR volume and the MTVs of ^18^F-FDG (A), ^11^C-MET (B), ^18^F-FLT (C), and ^18^F-FMISO (D), between the Gd-T1WI volume and the MTVs of ^18^F-FDG (E), ^11^C-MET (F), ^18^F-FLT (G), and ^18^F-FMISO (H), and between the DWI volume and the MTVs of ^18^F-FDG (I), ^11^C-MET (J), ^18^F-FLT (K), and ^18^F-FMISO (L) for each of the 4 glioma subtypes. Lines within the boxes indicate the average, boxes represent standard deviation, and whiskers denote minimum–maximum.

The MTVs of 4 PET tracers were smaller than that of the FLAIR volumes. No significant differences were observed among the glioma subtypes for comparisons between ^18^F-FDG MTVs and FLAIR volumes (Figure 3A) or between ^11^C-MET MTVs and FLAIR volumes (Figure 3B). Comparisons between ^18^F-FLT MTVs and FLAIR volumes revealed significant differences between GBM (0.49 ± 0.30) and both Mut (0.16 ± 0.16, *P* = .021) and Codel (0.15 ± 0.13, *P* = .004; Figure 3C). Comparisons between ^18^F-FMISO MTVs and FLAIR volumes revealed significant differences between Mut (0.15 ± 0.13) and GBM (0.36 ± 0.22, *P* = .003; Figure 3D).

##### Correlations Among Glioma Subtypes with the Comparison Between MTVs of 4 PET Tracers and Gd-T1WI Volumes. 

The MTVs of 4 PET tracers were much larger than the Gd-T1WI volumes for Mut, Codel, and Wt. For GBM, the MTVs of 4 PET tracers were similar or slightly larger than the Gd-T1WI volumes. Comparisons between ^18^F-FDG MTVs and Gd-T1WI volumes revealed a significant difference was identified between Mut (5.08 ± 4.85) and Wt (21.15 ± 21.80, *P* = .002). Significant differences were observed between GBM (0.97 ± 0.36) and both Codel (9.76 ± 16.95, *P* = .039) and Wt (21.15 ± 21.80, *P* < .001; Figure 3E). Regarding the comparisons between ^11^C-MET MTVs and Gd-T1WI volumes, there were significant differences between GBM (1.93 ± 1.60) and both Codel (21.41 ± 28.24, *P* = .005) and Wt (13.67 ± 18.35, *P* < .001; Figure 3F). Furthermore, comparisons between ^18^F-FLT MTVs and Gd-T1WI volumes revealed significant differences between Wt (21.21 ± 24.19) and Mut (4.51 ± 3.10), Codel (2.81 ± 4.47), or GBM (1.36 ± 0.56) (*P* < .001 for all; Figure 3G). Additionally, comparisons between ^18^F-FMISO MTVs and Gd-T1WI volumes demonstrated significant differences between Wt (12.70 ± 11.85) and Mut (4.78 ± 4.64, *P* = .008), Codel (2.29 ± 2.84, *P* = .005), or GBM (1.14 ± 0.66, *P* < .001; Figure 3H).

##### Correlation Among Glioma Subtypes with the Comparison Between MTVs of 4 PET Tracers and DWI Volumes. 

The ^11^C-MET, ^18^F-FLT, and ^18^F-FMISO MTVs were larger than the DWI volumes. ^18^F-FDG MTVs were similar or slightly lesser than the DWI volumes. For comparison between the volumes of DWI and MTVs of ^18^F-FDG, ^11^C-MET, or ^18^F-FLT tracers, there were no significant differences among the glioma subtypes (Figure 3I, J, and K). Comparison between MTV of ^18^F-FMISO and the volume of DWI indicated significant differences between Mut (0.34 ± 0.39) and Wt (1.45 ± 1.10, *P* = .046) or GBM (1.30 ± 0.69, *P* = .001; Figure 3L;Supplementary Table 3)

### Illustrative Cases

**Figure 4.**
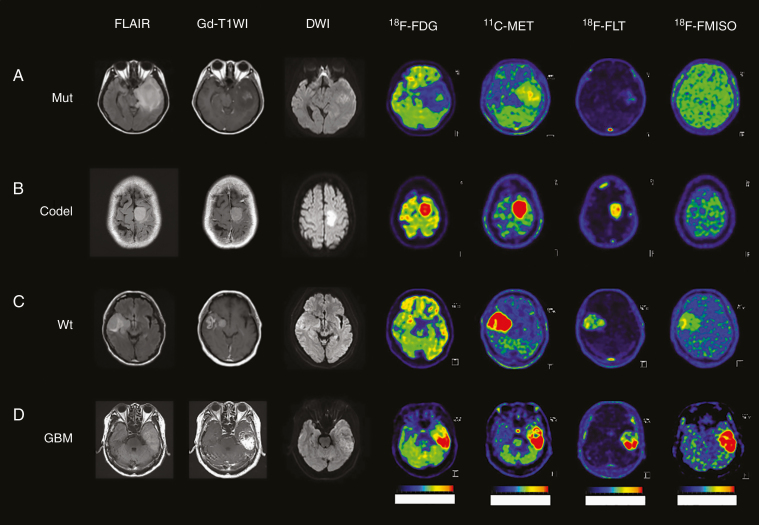
MRI (FLAIR, Gd-T1WI, and DWI) and PET images (^18^F-FDG, ^11^C-MET, ^18^F-FLT, and ^18^F-FMISO) in representative glioma patients. (A) A 29-year-old female patient in Mut subtype with the accumulation of ^11^C-MET and ^18^F-FLT. (B) A 38-year-old male patient in Codel subtype with high accumulation of ^11^C-MET, and slight accumulation of ^18^F-FLT. (C) A 58-year-old male patient in Wt subtype with the accumulation of ^11^C-MET and higher accumulation of ^18^F-FLT and ^18^F-FMISO than Mut. (D) A 69-year-old male patient with GBM with the highest accumulation of ^11^C-MET, ^18^F-FLT, and ^18^F-FMISO.

Figure 4 shows MRI and 4 PET images with the characteristics of each glioma subtype.

The comparative analyses revealed that Mut and Codel could be distinguished by ^11^C-MET. Since Codel exhibited a high ^11^C-MET accumulation, it was possible to distinguish Mut from Codel using the ^11^C-MET TNR cutoff (3.614). A 29-year-old female patient with Mut subtype (AA *IDH*-mut, 1p/19q noncodeletion) detected the accumulation of ^11^C-MET (SUV; 4.25, TNR; 2.891) and ^18^F-FLT (SUV; 0.59, TNR; 3.105) (Figure 4A). A 38-year-old female patient with Codel subtype (AA *IDH*-mut, 1p/19q codeletion) detected the higher accumulation of ^11^C-MET (SUV; 8.54, TNR; 6.672) than the cutoff value of ^11^C-MET (3.614) and the accumulation of ^18^F-FLT (SUV; 0.63, TNR; 3.316) (Figure 4B). Mut could be distinguished from Wt using the ^18^F-FLT TNR cutoff value (3.434). A 58-year-old male with the Wt subtype (AA *IDH*-wt) presented with a higher accumulation of ^18^F-FLT (SUV; 1.52, TNR; 5.846) than the cutoff value of ^18^F-FLT (3.434) and accumulation of ^11^C-MET (SUV; 10.6, TNR; 10.291). Therefore, ^18^F-FLT TNR could be used to diagnose this case as Wt (Figure 4C). A 69-year-old male patient with GBM presented with high accumulation of ^18^F-FDG (TNR; 4.376, MTV; 14.543 cm^3^), ^11^C-MET (TNR; 6.467, MTV; 16.833 cm^3^), ^18^F-FLT (TNR; 44.556, MTV; 15.078 cm^3^), and ^18^F-FMISO (TBR; 4.425, MTV; 13.514 cm^3^). These results demonstrated that the cutoff of TNR for ^18^F-FDG (2.127), ^11^C-MET (4.424), and ^18^F-FLT (6.455) and TBR for ^18^F-FMISO (1.760) and the cutoff of MTV for ^18^F-FDG (2.213), ^18^F-FLT (3.480), and ^18^F-FMISO (1.760) could distinguish between Mut and GBM. The cutoff of TNR for ^18^F-FLT (6.389) and TBR for ^18^F-FMISO (1.875) and the cutoff of MTV for ^18^F-FLT (5.627) could distinguish between Codel and GBM. The cutoff of TNR for ^11^C-MET (4.327) and ^18^F-FLT (7.563) and TBR for ^18^F-FMISO (1.612) could distinguish between Wt and GBM. Considering these results, case D was diagnosed as GBM (Figure 4D).

## Discussion

PET uses radiotracers to achieve metabolic and molecular imaging and, in combination with MRI, can provide useful information that may improve the diagnostic accuracy of brain tumors.^16,17^ One PET tracer is suitable for assessing related metabolism, but not for others. Therefore, the only approach which allows the simultaneous evaluation of various metabolites is using multiple PET tracers. Previous reports evaluating multiple PET tracers were based on systematic reviews based on the meta-analyses of published studies, and few reports evaluated multiple PET tracers used in the same patient.^18–20^ Furthermore, no report to date has evaluated the utility of multiple PET tracers including ^18^F-FMISO. The systematic reviews of published meta-analyses related to PET were based on the glioma classification according to WHO grades II, III, and IV and did not conform to the 2016 WHO classification of gliomas. This is the first report examining the interrelationship between 4 glioma subtypes based on the 2016 WHO classification and multiple PET tracers. PET tracer guidelines have been recently revised to provide joint practice guidelines and procedure standards for uniform, high-quality diagnostic accuracy imaging by the Working Group for Response Assessment in Neurooncology with PET.^17^  ^18^F-FDG is the most commonly used PET tracer in oncology. The present study results showed that ^18^F-FDG could distinguish between WHO grade II and IV gliomas, but ^18^F-FDG had the lowest sensitivity and specificity among the 4 PET tracers. Optimal quantitative thresholds and visual analysis criteria have not been established for the definitive differentiation of glioma grade based on ^18^F-FDG PET alone.^21^

Regarding amino acid PET tracers, especially, ^11^C-MET and ^18^F-FET are preferred over ^18^F-FDG due to the higher sensitivity.^17^ The present study revealed that the accumulation of ^11^C-MET increased in parallel with higher WHO malignancy grades. Although higher 11C-MET accumulations were observed in both OD and AO in previous studies,^22^  ^11^C-MET could not significantly differentiate between Mut and Codel at this time. Some reasons should be considered that there were relatively fewer patients with glioma subtypes other than GBM *IDH*-wt. Mut is composed of DA *IDH*-mut and AA *IDH*-mut, and Code is composed of OD and AO. Hence, AA and AO with higher malignancy are included in their respective subtypes; therefore, the accumulation of MET may have been high, and the difference between Mut and Codel may no longer be recognized. We would reanalyze the present study cohort with the addition of more patients and more extensive analysis between Mut and Codel in future investigations.

We previously reported that ^18^F-FLT could distinguish gliomas based on the 2007 WHO classification and that the ^18^F-FLT accumulation exhibited a strong correlation with the histopathologic proliferation marker Ki-67.^9^ Therefore, ^18^F-FLT is considered as a suitable tracer for evaluating tumor proliferation. However, careful consideration should be given to increased ^18^F-FLT accumulation related to its leakage from tumor vessels in brain tumors with a disrupted blood–brain barrier (BBB),^23,24^ and tumor blood flow.^25^ The 2016 WHO classification of gliomas is based on the *IDH* mutation status. The *IDH* mutation status was reported to be associated with tumor proliferation and prognosis in lower-grade gliomas.^26^ Takei *et al.* reported that ^11^C-MET could be used to differentiate between AA *IDH*-mut and AA *IDH*-wt and between GBM *IDH*-mut and GBM *IDH*-wt.^22^ No report to date has compared the association of ^18^F-FLT accumulation with tumor prognosis or *IDH* mutation status. In the present study, we first demonstrated that the comparison between ^18^F-FLT MTV and Gd-T1WI volume could be used to distinguish between Mut and Wt. In other words, it is suggested that Wt has a wider accumulation region of ^18^F-FLT than the enhancement region on Gd-T1WI compared with Mut.

^18^F-FMISO is a nitroimidazole derivative that is exclusively trapped in hypoxic cells. GBM presents with necrosis and hypoxic environment, whereas lower-grade gliomas do not develop necrosis; therefore, ^18^F-FMISO is more likely to accumulate in the hypoxic GBM environment.^11^ The present study results also suggest that ^18^F-FMISO can differentiate GBM from lower-grade gliomas. In the GBM microenvironment where hypoxia has progressed, hypoxia-inducible factor 1α (HIF1α) associated with hypoxia is activated. Most of GBM leads to upregulating HIF1α.^27^ We previously reported that the accumulation of ^18^F-FMISO was significantly correlated with the expression of vascular endothelial growth factor related to HIF1α.^11^ Therefore, it is reasonable to assume that the accumulation of ^18^F-FMISO would be high in patients with GBM. MTV of ^18^F-FMISO could be distinguished GBM *IDH*-wt from GBM *IDH*-mut, but ^18^F-FMISO accumulation alone cannot distinguish these subtypes. A recent report showed that not only hypoxia-related signaling pathways but also transforming growth factor β might be related to gliomas with *IDH*-wt.^28^ The comparison between ^18^F-FMISO MTV and Gd-T1WI volume or DWI volume could be used to distinguish between Mut and Wt. Gd-T1WI is related to the permeability of gadolinium, while DWI reflects on cell density. Because the ^18^F-FMISO MTV evaluates a wider area the Gd-T1WI and DWI volumes, ^18^F-FMISO in Wt might evaluate active tumor cell lesions, under hypoxia, and various other conditions. More evidence based on further investigation of larger cohorts is needed to confirm that ^18^F-FMISO can be used to differentiate between *IDH*-wt and *IDH*-mut gliomas.

The present study has several limitations. First limitation is that the metabolism of gliomas exhibiting various molecular changes could not be evaluated using only one PET tracer. In the present study, using 4 PET tracers that could assess different metabolic pathways allowed us to classify the study patients according to the 2016 WHO glioma classification, even though not all metabolic pathways could be evaluated. Codel and Wt could not be distinguished; however, these cases can generally be discriminated by comparing ^18^F-FMISO and MRI, and further examination using other tracers remains necessary. A second limitation was that few patients with Mut, Codel, and Wt were included in this study. The glioma subtypes were distributed non-normally and not homoscedastically. Therefore, it was Scheffe's multiple comparison procedure following the Kruskal–Wallis test was used for statistical analyses. The distribution can converge to a normal distribution by securing a greater number of cases; however, this will take time with a single center. The utility of multiple PET tracers in a greater number of patients across multiple institutions should be investigated.

## Conclusion

This is the first study examining the relationship between glioma classification based on the 2016 WHO classification and multiple PET tracers evaluating different metabolic pathways. We suggest that all PET tracers using ^18^F-FDG, ^11^C-MET, ^18^F-FLT, and ^18^F-FMISO are useful for the preoperative differential diagnosis of gliomas according to the 2016 WHO classification.

## Supplementary Material

vdaa172_suppl_Supplementary_Table_1

vdaa172_suppl_Supplementary_Table_2

vdaa172_suppl_Supplementary_Table_3

vdaa172_suppl_Supplementary_File_1

## Notes

**Authorship Statement.** K.M. designed and performed research; collected, analyzed, and interpreted data; and wrote the manuscript; T.O., D.O., and T.H. performed research; collected data; K.S. and A.S., performed research; histopathological and molecular analysis; N.K. helped with statistical analyses; Y.Y., and Y.N. performed research; analyzed PET data; T.T. supervised the study.
