# Supplementary material for: Multiple positron emission tomography tracers for use in the classification of gliomas according to the 2016 World Health Organization criteria
Source: Neurooncol Adv. 2020 Dec 7;3(1):vdaa172. doi: 10.1093/noajnl/vdaa172 (PMC7920529; doi:10.1093/noajnl/vdaa172)
Supplement: vdaa172_suppl_Supplementary_Table_1 [file vdaa172_suppl_supplementary_table_1.doc]

**Supplementary Table 1**

Volumes of FLAIR, Gd-T1WI, and DWI of the for four glioma subtypes

|  | | **MRI** | | |
| --- | --- | --- | --- | --- |
|  |  | **FLAIR** | **Gd-T1WI** | **DWI** |
| **Mut  vs  Codel** | **Cutoff Value** | 14.414 | 0.484 | 1.568 |
|  | **AUC** | 0.734 | 0.659 | 0.523 |
|  | **Sensitivity** | 0.864 | 0.773 | 0.546 |
|  | **Specificity** | 0.643 | 0.500 | 0.571 |
|  | **OR** | 11.400 | 3.400 | 1.600 |
|  | **95% CI** | 0.550 - 0.918 | 0.472 - 0.846 | 0.318 - 0.727 |
|  | ***p* value** | 0.671 | 0.969 | 0.974 |
| **Mut  vs  Wt** | **Cutoff Value** | 14.414 | 0.041 | 2.264 |
|  | **AUC** | 0.630 | 0.536 | 0.584 |
|  | **Sensitivity** | 0.864 | 0.500 | 0.455 |
|  | **Specificity** | 0.429 | 0.786 | 0.786 |
|  | **OR** | 4.750 | 3.667 | 3.056 |
|  | **95% CI** | 0.437 - 0.823 | 0.345 - 0.727 | 0.391 - 0.778 |
|  | ***p* value** | 0.821 | 0.999 | 0.979 |
| **Mut  vs  GBM** | **Cutoff Value** | **12.549** | **1.580** | 2.960 |
|  | **AUC** | **0.851** | **0.973** | 0.738 |
|  | **Sensitivity** | **0.818** | **0.909** | 0.682 |
|  | **Specificity** | **0.825** | **0.921** | 0.746 |
|  | **OR** | **21.273** | **116.000** | 6.295 |
|  | **95% CI** | **0.755 - 0.947** | **0.943 - 1.005** | 0.607 - 0.870 |
|  | ***p* value** | **0.035** | **< 0.001** | 0.127 |
| **Codel  vs  Wt** | **Cutoff Value** | 7.950 | 0.488 | 1.961 |
|  | **AUC** | 0.628 | 0.582 | 0.602 |
|  | **Sensitivity** | 0.857 | 0.500 | 0.500 |
|  | **Specificity** | 0.500 | 0.857 | 0.786 |
|  | **OR** | 6.000 | 6.000 | 3.667 |
|  | **95% CI** | 0.407 - 0.848 | 0.348 - 0.815 | 0.384 - 0.820 |
|  | ***p* value** | 0.995 | 0.967 | 0.881 |
| **Codel  vs  GBM** | **Cutoff Value** | 15.861 | **3.007** | 2.996 |
|  | **AUC** | 0.598 | **0.861** | 0.692 |
|  | **Sensitivity** | 0.500 | **0.857** | 0.714 |
|  | **Specificity** | 0.746 | **0.794** | 0.746 |
|  | **OR** | 2.938 | **23.077** | 7.344 |
|  | **95% CI** | 0.416 - 0.779 | **0.731 - 0.991** | 0.500 - 0.883 |
|  | ***p* value** | 0.771 | **0.036** | 0.537 |
| **Wt  vs  GBM** | **Cutoff Value** | 18.813 | **1.340** | 1.926 |
|  | **AUC** | 0.739 | **0.988** | 0.830 |
|  | **Sensitivity** | 0.786 | **1.000** | 0.786 |
|  | **Specificity** | 0.667 | **0.937** | 0.810 |
|  | **OR** | 7.333 | **191.750** | 15.583 |
|  | **95% CI** | 0.571 - 0.908 | **0.969 - 1.006** | 0.696 - 0.964 |
|  | ***p* value** | 0.585 | **0.006** | 0.101 |
