# Supplementary material for: Multiple positron emission tomography tracers for use in the classification of gliomas according to the 2016 World Health Organization criteria
Source: Neurooncol Adv. 2020 Dec 7;3(1):vdaa172. doi: 10.1093/noajnl/vdaa172 (PMC7920529; doi:10.1093/noajnl/vdaa172)
Supplement: vdaa172_suppl_Supplementary_Table_2 [file vdaa172_suppl_supplementary_table_2.doc]

| **Supplementary Table 2**  SUVs of four PET tracers, TNRs of three PET tracers, TBR of ^18^F-FMISO, and MTVs of four PET tracers for the four glioma subtypes | | | | | | | | | | | | | |
| --- | --- | --- | --- | --- | --- | --- | --- | --- | --- | --- | --- | --- | --- |
|  | | ^18^F-FDG | | | ^11^C-MET | | | ^18^F-FLT | | | ^18^F-FMISO | | |
|  |  | SUV | TNR | MTV | SUV | TNR | MTV | SUV | TNR | MTV | SUV | TBR | MTV |
| Mut  vs  Codel | Cutoff Value | 9.170 | 2.343 | 2.213 | 3.690 | 3.614 | 9.297 | 0.590 | 3.786 | 1.515 | 1.770 | 1.561 | 1.027 |
|  | AUC | 0.533 | 0.701 | 0.656 | 0.695 | 0.711 | 0.773 | 0.641 | 0.630 | 0.578 | 0.614 | 0.573 | 0.654 |
|  | Sensitivity | 0.727 | 0.727 | 0.773 | 0.546 | 0.682 | 0.864 | 0.546 | 0.682 | 0.636 | 0.636 | 0.727 | 0.682 |
|  | Specificity | 0.571 | 0.714 | 0.571 | 0.857 | 0.643 | 0.643 | 0.857 | 0.643 | 0.571 | 0.571 | 0.500 | 0.714 |
|  | OR | 3.556 | 6.667 | 4.533 | 7.200 | 3.857 | 15.200 | 7.200 | 3.857 | 2.333 | 2.333 | 2.667 | 4.821 |
|  | 95% CI | 0.320 – 0.745 | 0.513 – 0.890 | 0.456 – 0.856 | 0.522 – 0.868 | 0.540 – 0.882 | 0.592 – 0.954 | 0.456 – 0.826 | 0.440 – 0.820 | 0.377 – 0.779 | 0.403 – 0.825 | 0.366 – 0.781 | 0.462 – 0.846 |
|  | *p* value | 0.999 | 0.833 | 0.879 | 0.401 | 0.360 | 0.152 | 0.999 | 0.991 | 0.991 | 0.865 | 0.940 | 0.581 |
| Mut  vs  Wt | Cutoff Value | 10.140 | 1.689 | 2.213 | 2.480 | 3.614 | 3.137 | 0.530 | 3.434 | 1.515 | 1.650 | 1.487 | 1.027 |
|  | AUC | 0.513 | 0.513 | 0.588 | 0.549 | 0.584 | 0.659 | 0.645 | 0.734 | 0.672 | 0.550 | 0.541 | 0.659 |
|  | Sensitivity | 0.591 | 0.455 | 0.773 | 0.410 | 0.682 | 0.546 | 0.500 | 0.636 | 0.636 | 0.818 | 0.546 | 0.682 |
|  | Specificity | 0.571 | 0.643 | 0.500 | 0.857 | 0.500 | 0.714 | 0.857 | 0.786 | 0.643 | 0.357 | 0.643 | 0.714 |
|  | OR | 1.926 | 1.500 | 3.400 | 4.154 | 2.143 | 3.000 | 6.000 | 6.417 | 3.150 | 2.500 | 2.160 | 5.357 |
|  | 95% CI | 0.301 – 0.725 | 0.314 – 0.712 | 0.737 – 0.802 | 0.353 – 0.745 | 0.392 – 0.777 | 0.456 – 0.863 | 0.463 – 0.826 | 0.568 – 0.900 | 0.484 – 0.860 | 0.337 – 0.764 | 0.339 – 0.742 | 0.468 – 0.851 |
|  | *p* value | 0.840 | 0.793 | 0.365 | 0.994 | 0.949 | 0.330 | 0.978 | 0.791 | 0.812 | 0.999 | 1.000 | 0.830 |
| Mut  vs  GBM | Cutoff Value | 10.910 | **2.127** | **2.213** | **4.510** | **4.424** | 8.001 | **1.480** | **6.455** | **3.480** | **2.160** | **1.760** | **3.408** |
|  | AUC | 0.583 | **0.757** | **0.850** | **0.746** | **0.840** | 0.844 | **0.940** | **0.970** | **0.895** | **0.916** | **0.949** | **0.910** |
|  | Sensitivity | 0.682 | **0.682** | **0.773** | **0.682** | **0.773** | 0.773 | **0.909** | **0.909** | **0.818** | **0.909** | **0.910** | **0.864** |
|  | Specificity | 0.603 | **0.730** | **0.841** | **0.698** | **0.746** | 0.778 | **0.921** | **0.952** | **0.841** | **0.810** | **0.889** | **0.810** |
|  | OR | 3.257 | **2.798** | **18.020** | **4.962** | **9.988** | 13.357 | **116.000** | **200.000** | **22.050** | **42.500** | **80.000** | **26.389** |
|  | 95% CI | 0.439 – 0.728 | **0.649 – 0.865** | **0.752 – 0.948** | **0.612 – 0.881** | **0.743 – 0.936** | 0.752 – 0.936 | **0.867 – 1.014** | **0.936 – 1.005** | **0.813 – 0.977** | **0.856 – 0.975** | **0.904 – 0.994** | **0.841 – 0.977** |
|  | *p* value | 0.865 | **0.027** | **0.010** | **0.003** | **< 0.001** | 0.164 | **< 0.001** | **< 0.001** | **0.001** | **< 0.001** | **< 0.001** | **< 0.001** |
| Codel  vs  Wt | Cutoff Value | 9.090 | 2.359 | 3.026 | 3.850 | 4.426 | 10.277 | 0.790 | 4.476 | 2.265 | 1.970 | 1.490 | 1.766 |
|  | AUC | 0.505 | 0.648 | 0.525 | 0.653 | 0.648 | 0.539 | 0.503 | 0.592 | 0.569 | 0.617 | 0.594 | 0.544 |
|  | Sensitivity | 0.571 | 0.714 | 0.857 | 0.857 | 0.571 | 0.643 | 0.643 | 0.571 | 0.571 | 0.429 | 0.643 | 0.571 |
|  | Specificity | 0.571 | 0.714 | 0.357 | 0.500 | 0.714 | 0.571 | 0.429 | 0.571 | 0.500 | 0.857 | 0.643 | 0.571 |
|  | OR | 1.778 | 6.250 | 2.133 | 6.000 | 3.333 | 2.560 | 1.350 | 1.778 | 1.333 | 4.500 | 3.240 | 2.133 |
|  | 95% CI | 0.280 – 0.731 | 0.426 – 0.870 | 0.282 – 0.761 | 0.441 – 0.865 | 0.438 – 0.858 | 0.296 – 0.781 | 0.278 – 0.728 | 0.374 – 0.810 | 0.347 – 0.790 | 0.400 – 0.835 | 0.371 – 0.818 | 0.314 – 0.774 |
|  | *p* value | 0.931 | 0.999 | 0.857 | 0.638 | 0.756 | 0.982 | 0.996 | 0.941 | 0.950 | 0.848 | 0.961 | 0.982 |
| Codel  vs  GBM | Cutoff Value | 9.090 | 3.256 | 4.276 | 5.260 | 4.916 | 12.074 | **1.500** | **6.389** | **5.627** | **2.520** | **1.875** | 3.712 |
|  | AUC | 0.605 | 0.603 | 0.679 | 0.596 | 0.684 | 0.566 | **0.973** | **0.959** | **0.821** | **0.828** | **0.893** | 0.809 |
|  | Sensitivity | 0.571 | 0.786 | 0.571 | 0.643 | 0.643 | 0.643 | **0.929** | **0.857** | **0.857** | **0.857** | **0.857** | 0.786 |
|  | Specificity | 0.730 | 0.444 | 0.714 | 0.603 | 0.667 | 0.492 | **0.905** | **0.952** | **0.730** | **0.698** | **0.825** | 0.810 |
|  | OR | 3.608 | 2.933 | 3.185 | 2.736 | 3.600 | 1.540 | **123.500** | **120.000** | **14.824** | **13.895** | **28.364** | 22.917 |
|  | 95% CI | 0.421 – 0.790 | 0.460 – 0.751 | 0.524 – 0.850 | 0.437 – 0.756 | 0.527 – 0.840 | 0.379 – 0.754 | **0.946 – 1.003** | **0.914 – 1.004** | **0.689 – 0.953** | **0.711 – 0.946** | **0.791 – 0.995** | 0.659 – 0.958 |
|  | *p* value | 0.967 | 0.518 | 0.287 | 0.660 | 0.175 | 0.897 | **< 0.001** | **< 0.001** | **0.031** | **< 0.001** | **< 0.001** | 0.298 |
| Wt  vs  GBM | Cutoff Value | 10.090 | 2.053 | 2.583 | **5.270** | **4.327** | 6.654 | **1.220** | **7.563** | 3.994 | **2.070** | **1.612** | 3.469 |
|  | AUC | 0.557 | 0.727 | 0.691 | **0.755** | **0.806** | 0.592 | **0.916** | **0.881** | 0.712 | **0.918** | **0.947** | 0.771 |
|  | Sensitivity | 0.571 | 0.714 | 0.571 | **0.786** | **0.714** | 0.571 | **0.857** | **0.786** | 0.643 | **0.929** | **0.857** | 0.786 |
|  | Specificity | 0.651 | 0.778 | 0.825 | **0.587** | **0.794** | 0.810 | **0.984** | **0.905** | 0.810 | **0.841** | **0.937** | 0.810 |
|  | OR | 2.485 | 8.750 | 7.272 | **5.218** | **9.615** | 6.133 | **372.000** | **34.833** | 7.050 | **68.900** | **88.500** | 15.278 |
|  | 95% CI | 0.359 – 0.755 | 0.572 – 0.882 | 0.482 – 0.900 | **0.608 – 0.902** | **0.680 – 0.933** | 0.361 – 0.822 | **0.803 – 1.028** | **0.766 – 0.996** | 0.526 – 0.897 | **0.855 – 0.981** | **0.889 – 1.006** | 0.605 – 0.937 |
|  | *p* value | 0.990 | 0.576 | 0.874 | **0.040** | **0.006** | 0.996 | **0.003** | **< 0.001** | 0.164 | **< 0.001** | **< 0.001** | 0.115 |
