# Supplementary material for: Multiple positron emission tomography tracers for use in the classification of gliomas according to the 2016 World Health Organization criteria
Source: Neurooncol Adv. 2020 Dec 7;3(1):vdaa172. doi: 10.1093/noajnl/vdaa172 (PMC7920529; doi:10.1093/noajnl/vdaa172)
Supplement: vdaa172_suppl_Supplementary_Table_3 [file vdaa172_suppl_supplementary_table_3.doc]

| **Supplementary Table 3**  Correlation of glioma subtypes with the comparison between MTVs of four PET tracers and volumes of MRI | | | | | | | | | | | | | |
| --- | --- | --- | --- | --- | --- | --- | --- | --- | --- | --- | --- | --- | --- |
|  | | ^18^F-FDG | | | ^11^C-MET | | | ^18^F-FLT | | | ^18^F-FMISO | | |
|  |  | / FLAIR | / Gd-T1WI | / DWI | / FLAIR | / Gd-T1WI | / DWI | / FLAIR | / Gd-T1WI | / DWI | / FLAIR | / Gd-T1WI | / DWI |
| Mut  vs  Codel | Cutoff Value | 0.144 | 14.032 | 0.625 | 0.600 | 4.282 | 0.986 | 0.220 | 2.101 | 0.233 | 0.093 | 2.032 | 0.587 |
|  | AUC | 0.587 | 0.535 | 0.561 | 0.608 | 0.546 | 0.537 | 0.591 | 0.688 | 0.556 | 0.528 | 0.528 | 0.749 |
|  | Sensitivity | 0.773 | 0.917 | 0.611 | 0.546 | 0.917 | 0.556 | 0.500 | 0.833 | 0.611 | 0.409 | 0.667 | 0.833 |
|  | Specificity | 0.462 | 0.417 | 0.692 | 0.769 | 0.500 | 0.846 | 0.769 | 0.667 | 0.539 | 0.692 | 0.583 | 0.692 |
|  | OR | 2.914 | 7.857 | 2.357 | 4.000 | 4.500 | 4.286 | 3.333 | 10.000 | 1.400 | 1.558 | 2.800 | 13.125 |
|  | 95% CI | 0.387 – 0.788 | 0.281 – 0.788 | 0.333 – 0.789 | 0.412 – 0.805 | 0.235 – 0.856 | 0.289 – 0.785 | 0.401 – 0.781 | 0.445 – 0.930 | 0.332 – 0.779 | 0.323 – 0.733 | 0.273 – 0.783 | 0.550 – 0.948 |
|  | *p* value | 0.939 | 0.681 | 0.275 | 0.773 | 0.694 | 0.966 | 0.819 | 0.969 | 0.945 | 0.898 | 0.999 | 0.135 |
| Mut  vs  Wt | Cutoff Value | 0.174 | **14.032** | 0.888 | 0.887 | 15.437 | 0.986 | 0.668 | **10.328** | 0.902 | 0.093 | **5.908** | **0.732** |
|  | AUC | 0.513 | **0.712** | 0.648 | 0.565 | 0.582 | 0.719 | 0.649 | **0.652** | 0.744 | 0.542 | **0.742** | **0.889** |
|  | Sensitivity | 0.727 | **0.917** | 0.667 | 0.818 | 0.917 | 0.778 | 0.909 | **0.833** | 0.778 | 0.409 | **0.750** | **0.889** |
|  | Specificity | 0.500 | **0.636** | 0.750 | 0.500 | 0.636 | 0.667 | 0.500 | **0.636** | 0.750 | 0.714 | **0.727** | **0.833** |
|  | OR | 2.667 | **19.250** | 4.000 | 4.500 | 3.333 | 11.429 | 10.000 | **8.750** | 8.167 | 1.731 | **8.000** | **56.000** |
|  | 95% CI | 0.295 – 0.731 | **0.466 – 0.959** | 0.409 – 0.887 | 0.349 – 0.781 | 0.190 – 0.974 | 0.517 – 0.921 | 0.453 – 0.846 | **0.392 – 0.911** | 0.536 – 0.953 | 0.336 – 0.748 | **0.514 – 0.970** | **0.751 – 1.027** |
|  | *p* value | 1.000 | **0.002** | 0.368 | 0.300 | 0.219 | 0.181 | 0.318 | **< 0.001** | 0.136 | 0.378 | **0.005** | **0.046** |
| Mut  vs  GBM | Cutoff Value | 0.266 | 1.932 | 0.625 | 0.468 | 4.282 | 0.986 | **0.272** | **2.101** | 0.902 | **0.209** | 1.582 | **0.587** |
|  | AUC | 0.522 | 0.792 | 0.746 | 0.553 | 0.930 | 0.686 | **0.709** | **0.865** | 0.805 | **0.776** | 0.804 | **0.920** |
|  | Sensitivity | 0.500 | 0.667 | 0.611 | 0.818 | 0.833 | 0.556 | **0.636** | **0.833** | 0.778 | **0.727** | 0.750 | **0.833** |
|  | Specificity | 0.587 | 0.968 | 0.873 | 0.381 | 0.889 | 0.937 | **0.714** | **0.825** | 0.873 | **0.778** | 0.857 | **0.921** |
|  | OR | 1.423 | 61.000 | 10.607 | 2.769 | 82.500 | 17.857 | **4.375** | **23.636** | 21.000 | **9.333** | 18.000 | **78.000** |
|  | 95% CI | 0.363 – 0.678 | 0.604 – 0.980 | 0.603 – 0.890 | 0.418 – 0.687 | 0.846 – 1.013 | 0.503 – 0.869 | **0.564 – 0.853** | **0.721 – 1.009** | 0.657 – 0.952 | **0.652 – 0.899** | 0.627 – 0.981 | **0.833 – 1.006** |
|  | *p* value | 0.972 | 0.563 | 0.999 | 0.996 | 0.099 | 0.713 | **0.021** | **< 0.001** | 0.057 | **0.003** | 0.131 | **0.001** |
| Codel  vs  Wt | Cutoff Value | 0.513 | 17.591 | 0.859 | 0.865 | 15.410 | 1.281 | 0.208 | **2.086** | 0.804 | 0.161 | **3.618** | 0.826 |
|  | AUC | 0.539 | 0.621 | 0.622 | 0.522 | 0.600 | 0.708 | 0.703 | **0.667** | 0.673 | 0.506 | **0.621** | 0.677 |
|  | Sensitivity | 0.357 | 0.750 | 0.615 | 0.769 | 0.667 | 0.615 | 0.769 | **0.667** | 0.615 | 0.692 | **0.667** | 0.615 |
|  | Specificity | 0.643 | 0.636 | 0.667 | 0.500 | 0.546 | 0.667 | 0.643 | **0.727** | 0.750 | 0.429 | **0.727** | 0.833 |
|  | OR | 6.667 | 5.250 | 4.667 | 3.333 | 4.667 | 8.000 | 6.000 | **5.333** | 6.222 | 1.688 | **5.333** | 9.333 |
|  | 95% CI | 0.305 – 0.772 | 0.373 – 0.869 | 0.343 – 0.901 | 0.283 – 0.761 | 0.222 – 0.978 | 0.440 – 0.977 | 0.491 – 0.916 | **0.426 – 0.908** | 0.422 – 0.923 | 0.276 – 0.735 | **0.368 – 0.875** | 0.422 – 0.931 |
|  | *p* value | 0.966 | 0.051 | 0.999 | 0.891 | 0.819 | 0.143 | 0.093 | **< 0.001** | 0.460 | 0.856 | **0.008** | 0.951 |
| Codel  vs  GBM | Cutoff Value | 0.199 | **1.609** | 0.859 | 0.667 | **2.480** | 2.042 | **0.208** | 1.309 | 0.804 | 0.161 | 1.299 | 0.826 |
|  | AUC | 0.642 | **0.759** | 0.695 | 0.613 | **0.860** | 0.699 | **0.866** | 0.581 | 0.740 | 0.742 | 0.712 | 0.675 |
|  | Sensitivity | 0.539 | **0.667** | 0.615 | 0.769 | **0.750** | 0.385 | **0.769** | 0.667 | 0.615 | 0.692 | 0.667 | 0.615 |
|  | Specificity | 0.746 | **0.905** | 0.619 | 0.556 | **0.873** | 0.984 | **0.794** | 0.571 | 0.889 | 0.873 | 0.714 | 0.778 |
|  | OR | 3.427 | **19.000** | 5.281 | 4.167 | **11.500** | 6.500 | **12.821** | 2.667 | 18.667 | 15.469 | 5.000 | 8.191 |
|  | 95% CI | 0.451 – 0.833 | **0.549 – 0.969** | 0.485 – 0.906 | 0.459 – 0.767 | **0.722 – 0.998** | 0.517 – 0.881 | **0.770 – 0.962** | 0.348 – 0.813 | 0.510 – 0.971 | 0.563 – 0.921 | 0.507 – 0.916 | 0.440 – 0.910 |
|  | *p* value | 0.724 | **0.039** | 0.198 | 0.790 | **0.005** | 0.557 | **0.004** | 0.496 | 0.478 | 0.154 | 0.157 | 0.925 |
| Wt  vs  GBM | Cutoff Value | 0.146 | **1.812** | 0.991 | 0.910 | **8.915** | 2.986 | 0.291 | **3.603** | 0.967 | 0.166 | **2.655** | 1.076 |
|  | AUC | 0.574 | **0.827** | 0.541 | 0.625 | **0.731** | 0.537 | 0.543 | **0.795** | 0.504 | 0.666 | **0.859** | 0.509 |
|  | Sensitivity | 0.500 | **0.818** | 0.500 | 0.500 | **0.818** | 0.583 | 0.500 | **0.727** | 0.583 | 0.643 | **0.818** | 0.500 |
|  | Specificity | 0.794 | **0.968** | 0.778 | 0.746 | **0.968** | 0.825 | 0.698 | **0.952** | 0.651 | 0.857 | **0.968** | 0.778 |
|  | OR | 3.846 | **137.250** | 1.850 | 2.938 | **85.500** | 2.286 | 2.316 | **53.333** | 3.067 | 10.800 | **137.250** | 2.957 |
|  | 95% CI | 0.349 – 0.799 | **0.603 – 1.051** | 0.288 - 0.794 | 0.436 – 0.813 | **0.410 – 1.052** | 0.312 – 0.762 | 0.330 – 0.756 | **0.583 – 1.007** | 0.242 – 0.765 | 0.455 – 0.876 | **0.666 – 1.051** | 0.275 – 0.742 |
|  | *p* value | 0.976 | **< 0.001** | 0.289 | 0.251 | **< 0.001** | 0.424 | 0.976 | **< 0.001** | 0.963 | 0.674 | **< 0.001** | 0.999 |
