# Supplementary material for: Multiple positron emission tomography tracers for use in the classification of gliomas according to the 2016 World Health Organization criteria
Source: Neurooncol Adv. 2020 Dec 7;3(1):vdaa172. doi: 10.1093/noajnl/vdaa172 (PMC7920529; doi:10.1093/noajnl/vdaa172)
Supplement: vdaa172_suppl_Supplementary_File_1 [file vdaa172_suppl_supplementary_file_1.docx]

**Supplementary files**

**Materials and Methods**

*Patients*

This retrospective, single-center study complied with the precepts established by the Declaration of Helsinki and was approved by the Kagawa University Faculty of Medicine Human Subjects Ethics Committee (no. 2019-027). ^18^F-labeled fluoro-2-deoxyglucose (^18^F-FDG), ^11^C-methionine (^11^C-MET), ^18^F-fluorothymidine (^18^F-FLT), and ^18^F-fluoromisonidazole (^18^F-FMISO) were approved for use as PET tracers by the Kagawa University Faculty of Medicine Human Subjects Ethics Committee, and an informed written consent was obtained from all participants.

From April 2009 to March 2019, 130 patients underwent ^18^F-FDG, ^11^C-MET, ^18^F-FLT, and ^18^F-FMISO PET evaluation at Kagawa University Faculty of Medicine in Japan. We included 113 patients in the final diagnosis after excluding those who were not assessed by all four PET tracers, did not undergo histopathological and molecular analyses, and were diagnosed with not-otherwise-specified lesions (Table 1).^2^

According to the 2016 WHO criteria,^2^ tumors were classified as diffuse astrocytoma (DA) with isocitrate dehydrogenase *(IDH)1/2* mutation (mut) without 1p19q codeletion (DA *IDH-*mut), anaplastic astrocytoma (AA) with *IDH1/2*-mut without 1p19q codeletion (AA *IDH*-mut), oligodendroglioma (OD) with *IDH1/2*-mut and 1p19q codeletion, anaplastic oligodendroglioma (AO) with *IDH1/2*-mut and 1p19q codeletion, DA with *IDH1/2* wild type (wt) (DA *IDH-*wt), AA with *IDH1/2* wt (AA *IDH*-wt), glioblastoma multiforme (GBM) with *IDH1/2*-mut (GBM *IDH*-mut), and GBM with *IDH1/2* wt (GBM *IDH*-wt). In this study, tumors were divided into Mut, Codel, Wt, and GBM glioma subtypes and were evaluated as follows: Mut, DA *IDH*-mut and AA *IDH*-mut; Codel, OD and AO; Wt, DA *IDH*-wt and AA *IDH*-wt; and GBM, GBM *IDH*-mut and GBM *IDH*-wt. All included patients were orally informed with the details regarding the study and provided their informed consent.

*Histopathological and molecular analyses*

To reclassify the study cohort according to the 2016 WHO classification, the study patients were evaluated for *IDH*-mut and 1p19q codeletion. For *IDH*-mut status, *IDH*1^R132H^ protein expression was determined by immunohistochemistry using a monoclonal antibody (clone H09, 1:50; Dianova, Germany). In cases where immunostaining was not possible, *IDH1* (R132) and *IDH2* (R172) were directly sequenced using the Sanger method. The 1p19q codeletion status was analyzed by fluorescence *in situ* hybridization with locus-specific probes for 1p36 and 19q13.

*MRI and PET*

MRI was performed on a 3-T MAGNETOM Skyra (Siemens Healthcare, Erlangen, Germany) scanner. T2-weighted axial fluid-attenuated inversion recovery images (FLAIR) (repetition time/echo time, 10000/93 ms; slice, 5 mm; matrix, 224 × 320), gadolinium-contrast axial T1-weighted images (Gd-T1WI) (repetition time/echo time, 400/11 ms; slice, 5 mm; matrix, 230 × 384) and diffuse weighted imaging (DWI) (repetition time/echo time, 5300/69 ms; slice, 5 mm; matrix, 160 × 160; 1000 second/mm^2^ B-value) were acquired.

PET studies were performed using a Biograph mCT PET/CT scanner (Siemens Medical Solutions Knoxville, TN, USA). PET scans were acquired in the three-dimensional model, and PET images were reconstructed as described in our previous study [the simultaneous acquisition of 51 transverse images per field of view (FOV), with an intersection spacing of 3 mm, for a total axial FOV of 15 cm].^10^ PET radiotracers were produced using an HM-18 cyclotron (Sumitomo Heavy Industries, Tokyo, Japan). The radiochemical purity of ^11^C-MET,^12^ ^18^F-FLT,^13^ and ^18^F-FMISO^14^ were >95%. Transmission and regional emission images of the brain were obtained as described in our previous study.^10^ Fasting was initiated 6 h before all PET studies, and the examination schedule was as follows: MRI, including contrast examination, was performed on day 1, ^18^F-FMISO was performed on day 2, ^18^F-FLT was performed on day 3, and ^11^C-MET was performed on the morning of day 4, followed by ^18^F-FDG during the afternoon of day 4.

*Image analyses*

The uptake of ^18^F-FDG, ^11^C-MET, and ^18^F-FLT in brain tumors were semiquantitatively assessed by obtaining the standardized uptake values (SUVs). A region of interest around the hottest portion of each lesion was manually set by an observer. The maximum SUV (SUV_max_) was considered as the representative value for each tumor. The maximum tumor-to-normal ratio (TNR) was determined by dividing the tumor SUV_max_ by the mean SUV of the normal brain parenchyma (usually contralateral normal cerebral tissue excluding the ventricles). The uptake of ^18^F-FMISO in the brain tumor was semiquantitatively assessed by evaluating the SUV_max_. The ^18^F-FMISO PET images were converted into average venous blood concentration of ^18^F-FMISO to obtain the tumor-to-blood ratios (TBRs), allowing for a three-dimensional pixel-by-pixel calculation of the maximum TBR for SUV_max_. The tumor volumes were measured by performing a three-dimensional, threshold-based, volume-of-interest analysis of the hyperintensity on fluid-attenuated inversion recovery (FLAIR) images, hyperintensity on diffusion-weighted images (DWI), and contrast-enhanced lesions on gadolinium-enhanced T1-weighted images (Gd-T1WI). For PET studies, the cutoff values of 1.1 on the ^18^F-FDG TNR, 1.3 on the ^11^C-MET TNR, 1.3 on the ^18^F-FLT TNR, and 1.2 on the ^18^F-FMISO TBR were used to determine the metabolic tumor volume (MTV).^8,15^ The PET and MRI datasets were transferred to a Linux workstation, and coregistration of ^18^F-FDG/^11^C-MET/^18^F-FLT/^18^F-FMISO/MRI was performed using Dr. View/Linux, version R2.5 (AJS, Tokyo, Japan). Before the histopathological and molecular diagnoses, two radiologists (Y. Y. and Y. N.) analyzed the data to lower the risk of observer bias to the maximum extent possible.

*Statistical analysis*

The relationship of glioma subtypes with the volume on FLAIR, Gd-T1WI, and DWI; mean TNRs on ^18^F-FDG, ^11^C-MET, and ^18^F-FLT; mean TBR on ^18^F-FMISO; and MTV on four PET studies was examined. After conducting the Kruskal-Wallis test, a comparative analysis of TNRs and TBR, and the MTV of each PET tracer was performed following the Scheffe’s multiple comparison method to assess the diagnostic accuracy of PET tracers in distinguishing glioma subtypes. All parametric data were expressed as means ± SD. A *p* value of <0.05 was considered significant. The cutoff values for volumes on FLAIR, Gd-T1WI, and DWI; mean TNRs on ^18^F-FDG, ^11^C-MET, and ^18^F-FLT; mean TBR on ^18^F-FMISO; and MTVs on four PET studies in the receiver operating characteristic (ROC) curve, area under the curve (AUC), sensitivity, specificity, odds ratios (ORs), 95% confidence interval (CI), and *p* value by the log-rank test at the cutoff value were compared and examined between glioma subtypes. The cutoff values with the highest sensitivity and specificity were used in the analysis. The MTV of each PET tracer and the volume of each MRI were compared (MTV of PET divided by the volume of MRI) and examined. All statistical analyses were performed using the SPSS statistical software package (version 26; IBM).

**Results**

1. Volumes of FLAIR, Gd-T1WI, and DWI of the four glioma subtypes

In Supplementary Table 1, the FLAIR volumes significantly differed between Mut and GBM (cutoff value, 12.549 cm^3^; *p* = 0.035). The Gd-T1WI volumes significantly differed between GBM and Mut (cutoff value, 1.580 cm^3^; *p* < 0.001), Codel (cutoff value, 3.007 cm^3^; *p* = 0.036), and Wt (cutoff value, 1.340 cm^3^; *p* = 0.006). By contrast, there was no significant difference between the DWI volumes among the glioma subtypes. Meanwhile, significant differences were observed in the volumes of Gd-T1WI between the GBM subtypes and other glioma subtypes.

1. Correlation of glioma subtypes with SUVs of four PET tracers, TNRs of three PET tracers, TBR of ^18^F-FMISO, and MTVs of four PET tracers

Supplementary Table 2 summarizes cutoff values for SUVs of four PET tracers, TNRs of three PET tracers, TBR of ^18^F-FMISO, and MTVs of four PET tracers. The ROC curve, AUC, sensitivity, specificity, OR, 95% CI, and log-rank *p* value of the cutoff values were compared and examined among the four glioma subtypes. Significant differences were observed in the ^11^C-MET SUVs between GBM and Mut (cutoff value, 4.510; *p* = 0.003) or Wt (cutoff value, 5.270; *p* = 0.040). Significant differences were observed in the ^18^F-FLT SUVs between GBM and Mut (cutoff value, 1.480; *p* < 0.001), Codel (cutoff value, 1.500; *p* < 0.001), or Wt (cutoff value, 1.220; *p* = 0.003). Significant differences were noted in the ^18^F-FMISO SUVs between GBM and Mut (cutoff value, 2.160), Codel (cutoff value, 2.520), or Wt (cutoff value, 2.070) (*p* < 0.001 for all). Significant differences were noted in the ^18^F-FDG TNRs between Mut and GBM (cutoff value, 2.127; *p* = 0.027). Significant differences were found in the ^11^C-MET TNRs between GBM and Mut (cutoff value, 4.424; *p* < 0.001) or Wt (cutoff value, 4.327; *p* = 0.006). Significant differences were found in the ^18^F-FLT TNRs between GBM and Mut (cutoff value, 6.455), Codel (cutoff value, 6.389), or Wt (cutoff value, 7.563) (*p* < 0.001 for all). Significant differences were observed in the ^18^F-FMISO TBRs between GBM and Mut (cutoff value, 1.760), Codel (cutoff value, 1.875), or Wt (cutoff value, 1.612) (*p* < 0.001 for all). Significant differences were observed in the ^18^F-FDG MTVs between GBM and Mut (cutoff value, 2.213 cm^3^; *p* = 0.010). Meanwhile, no significant difference was observed in the ^11^C-MET MTVs among the glioma subtypes. By contrast, significant differences were noted in the ^18^F-FLT MTVs between the GBM and Mut (cutoff value, 3.480 cm^3^; *p* = 0.001) or Codel (cutoff value, 5.627 cm^3^; *p* = 0.031). Significant differences were also observed in the ^18^F-FMISO MTVs between GBM and Mut (cutoff value, 3.408 cm^3^; *p* < 0.001).

1. Correlation of glioma subtypes with the comparison between MTVs of four PET tracers and volumes of MRI

Supplementary Table 3 summarizes the cutoff values for the comparison between the volume of each MRI and MTVs of four PET tracers. The ROC curve, AUC, sensitivity, specificity, OR, 95% CI, and log-rank *p* value of the cutoff values were compared and examined among the four glioma subtypes. Significant differences were noted in the comparison between ^18^F-FLT MTVs and FLAIR volumes between GBM and Mut (cutoff value, 0.272; *p* = 0.021), and Codel (cutoff value, 0.208; *p* = 0.004). Significant differences were noted in the comparison between ^18^F-FMISO MTVs and FLAIR volumes between GBM and Mut (cutoff value, 0.209; *p* = 0.003). Significant differences were observed in the comparison between ^18^F-FDG MTVs and Gd-T1WI volumes between Mut and Wt (cutoff value, 14.032; *p* = 0.002) and between GBM and Codel (cutoff value, 1.609; *p* = 0.039) or Wt (cutoff value, 1.812; *p* < 0.001). Significant differences were observed in the comparison between ^11^C-MET MTVs and Gd-T1WI volumes between GBM and Codel (cutoff value, 2.480; *p* = 0.005) or Wt (cutoff value, 8.915; *p* < 0.001). Significant differences were found in the comparison between ^18^F-FLT MTVs and Gd-T1WI volumes between Wt and Mut (cutoff value, 10.328), Codel (cutoff value, 2.086), or GBM (cutoff value, 3.603) (*p* < 0.001 for all). Significant differences were found in the comparison between ^18^F-FMISO MTVs and Gd-T1WI volumes between Wt and Mut (cutoff value, 5.908), Codel (cutoff value, 3.618), or GBM (cutoff value, 2.655) (*p* < 0.001 for all). Meanwhile, no significant difference was found in the comparison between DWI volumes and ^18^F-FDG MTVs, ^11^C-MET MTVs, or 18F-FLT MTVs among the glioma subtypes. By contrast, significant differences were observed in the comparison between ^18^F-FMISO MTVs and DWI volumes between Mut and Wt (cutoff value, 0.732; *p* = 0.046) and GBM (cutoff value, 0.587; *p* = 0.001).

**Supplementary Tables legends**

Supplementary Table 1:

Volumes of FLAIR, Gd-T1WI, and DWI of the four glioma subtypes

The cutoff values for volumes of FLAIR, Gd-T1WI, and DWI in the ROC curve, AUC, sensitivity, specificity, OR, 95% CI, and *p* value by the log-rank test at the cutoff value were compared and examined among the four glioma subtypes. The comparison between Mut and Codel, Wt, or GBM; between Codel and Wt or GBM; and between Wt and GBM. Gd-T1WI volumes significantly differed between GBM and Mut, Codel, and Wt (*p* < 0.001 for all). The four glioma subtypes were Mut, Codel, Wt, and GBM (Mut, DA IDH-mut and AA IDH-mut; Codel, OD and AO; Wt, DA IDH-wt and AA IDH-wt; and GBM, GBM IDH-mut and GBM IDH-wt). DA, diffuse astrocytoma; OD, oligodendroglioma; AA, anaplastic astrocytoma; AO, anaplastic oligodendroglioma; GBM, glioblastoma multiforme; PET, positron emission tomography; IDH, isocitrate dehydrogenase; mut, mutation; wt, wild type; FLAIR, fluid-attenuated inversion recovery; Gd-T1WI, gadolinium contrast-enhanced axial T1-weighted images; DWI, diffusion-weighted imaging; ROC, receiver operating characteristic; AUC, area under the curve; OR, odds ratio; 95% CI, 95% confidence interval. Bold indicates a *p* value <0.05.

Supplementary Table 2:

SUVs of four PET tracers, TNRs of three PET tracers, TBR of ^18^F-FMISO, and MTVs of four PET tracers for four glioma subtypes

The cutoff values for SUVs of four PET tracers, TNRs of three PET tracers, TBR of ^18^F-FMISO, and MTVs of four PET tracers in the ROC curve, AUC, sensitivity, specificity, OR, 95% CI, and *p* value by the log-rank test at the cutoff value were compared and examined among the four glioma subtypes. A comparative analysis between Mut and Codel, Wt, or GBM; between Codel and Wt or GBM; and between Wt and GBM was performed. Significant differences were found in the TNRs of ^18^F-FLT and TBR of ^18^F-FMISO between GBM and Mut, Codel, or Wt (*p* < 0.001 for all). Significant differences were also found in the MTVs of ^18^F-FDG, ^18^F-FLT and ^18^F-FMISO between Mut and GBM (*p* < 0.05 for all). Significant differences were also found in the MTVs of ^18^F-FLT between Codel and GBM (*p* = 0.031). SUV, standardized uptake value; TNR, tumor-to-normal ratio; TBR, tumor-to-blood ratio; MTV, metabolic tumor volume. Bold indicates a *p* value <0.05.

Supplementary Table 3:

Correlation of glioma subtypes with the comparison between MTVs of four PET tracers and volumes of MRI

The cutoff values for the comparison between volume of each MRI and MTVs of four PET tracers in the ROC curve, AUC, sensitivity, specificity, OR, 95% CI, and *p* value by the log-rank test at the cutoff value were compared and examined among the four glioma subtypes. A comparative analysis between Mut and Codel, Wt, or GBM; between Codel and Wt or GBM; and between Wt and GBM was performed. Significant differences were noted in the comparison between MTVs of four PET tracers and Gd-T1WI volumes between Mut and GBM (*p* < 0.001 for all). Significant differences were noted in the comparison between MTVs of ^18^F-FLT or ^18^F-FMISO and Gd-T1WI volumes between Wt and Mut or Codel (*p* < 0.01 for all). Significant differences were also noted in the comparison between MTV of ^18^F-FMISO and DWI volume between Mut and Wt (*p* = 0.046), or Codel (*p* = 0.001). Bold indicates a *p* value <0.05.
